# Effect of Subantimicrobial Dose Doxycycline Treatment on Gingival Crevicular Fluid Levels of MMP-9 and MMP-13 in Periodontitis Stage 2, Grade B in Subjects with Type 2 Diabetes Mellitus

**DOI:** 10.1155/2020/2807259

**Published:** 2020-11-20

**Authors:** Mai S. Attia, Jazia A. Alblowi

**Affiliations:** ^1^Department of Oral Medicine, Periodontology, Diagnosis and Radiology, Faculty of Dental Medicine for Girls, Al-Azhar University (Girls Branch), Cairo, Egypt; ^2^Periodontology Department, Faculty of Dentistry, King Abdulaziz University, Jeddah, Saudi Arabia

## Abstract

The aim of this study was to investigate the effect of using subantimicrobial dose doxycycline as an adjunct in periodontitis stage 2, grade B in subjects with type 2 diabetes mellitus. A total of thirty patients were divided into the following two groups with reference to periodontitis, type 2 diabetes mellitus, and administration of the doxycycline drug: Group I: patients with periodontitis stage 2, grade B and type 2 diabetes mellitus who received SRP only. Group II: patients with periodontitis stage 2, grade B and type 2 diabetes mellitus who received SRP and doxycycline 20 mg. The following clinical measurements were recorded at baseline (prior to scaling and root planning) and after one and three months postoperatively: GI, PI, and PD with a periodontal calibrated probe. The levels of both MMP-9 and MMP-13, from 60 GCF samples, were analyzed by ELISA. Patients treated with SRP and doxycycline 20 mg showed a significant reduction of PD, PI, GI, MMP-9, and MMP-13 than patients who received SRP only. Improvements in parameters clinically and biochemically were observed following the adjunctive use of doxycycline subantimicrobial dose therapy for the management of stage 2, grade B periodontitis patients with type 2 diabetes mellitus.

## 1. Introduction

Periodontitis is a progressive multifactorial condition with accumulation of plaque and calculus and change in the subgingival microbiome ecology. This leads to a slow but progressive destruction of the periodontium [1].

Papapanou et al. [2] proposed a new and more specific periodontal disease classification. This classification involves four stages of periodontitis according to severity, complexity, extent, and distribution. In addition to the stages, three grades that reflect biological characteristics were also established. This study targeted patients with stage 2, grade B periodontitis [2].

Periodontitis as chronic inflammation causes the activation and excessive inflammatory mediators' secretion that are responsible for the destruction of connective tissue and bone. Among these are cytokines, prostaglandin E2 (PGE2), tumor necrosis factor alpha (TNF*α*), interleukins IL-1*β* and IL-6, matrix metalloproteinase (MMPs), receptor activator of nuclear factor kappa-B ligand (RANKL), regulatory T cell cytokines (IL-12 and IL-18), and chemokines [3].

As periodontitis, inflammation is also a feature of diabetes mellitus (DM), constituting the main link between the two diseases. In this way, DM is associated with high levels of inflammatory systemic markers [4]^,^ which contribute to micro- and macrovascular complications. It has been shown that chronic hyperglycemia activates different pathways that cause increased inflammation, oxidative stress, and apoptosis [5]. In these processes, elevated blood levels of IL-6 and TNF*α* would appear to be key elements, with the associated increase in acute phase proteins, such as C-reactive protein. Given the importance of inflammation in both diseases, the most widely accepted hypothesis postulates that DM could increase the periodontal tissue inflammatory response and periodontitis worsens diabetic control [6].

A wide variety of immune mediators are involved in the evolution of the mentioned diseases; examples of these mediators are matrix metalloproteinase-9 (MMP-9) and matrix metalloproteinase-13 (MMP-13). With respect to MMP-9, it is a gelatinase associated with the degradation of gelatin and type IV collagen and is identified in gingival crevicular fluid samples from patients with periodontitis [7]. MMP-9 is mainly synthesized by neutrophils, with increased production during inflammatory processes [8].

On the other hand, MMP-13 is a collagenase detected in fibroblasts, macrophages, osteoblasts, plasma cells and gingival epithelial cells. Previously, MMP-13 has been involved in the destruction of periodontal soft tissue and, together with MMP-9, has been involved in alveolar bone resorption and the breakdown of periodontal tissue [9, 10].

Nonsurgical periodontal therapy (NSPT) is the cornerstone of periodontal therapy and the first recommended approach for the control of periodontal infections [11]. However, SRP procedures do not eliminate all periodontopathic bacteria from the subgingival area, especially those in inaccessible areas such as furcations, furrows, concavities, and deep pockets. It seems that the effects of mechanical therapy could be increased using antimicrobial agents that further suppress the remaining pathogens [12].

Several drugs have been tried to modulate the host response, such as nonsteroidal anti-inflammatory drugs (NSAIDs), bisphosphonates, and the family of tetracycline compounds and their chemically modified analogs. However, until today, the subantimicrobial dose doxycycline (SDD) is the only medication that has been approved for host modulation in periodontal therapy [13].

SDD downregulates expression of main inflammatory cytokines (IL-1, IL-6, and tumor necrosis factor *α*) and prostaglandin E2. SDD can cause direct inhibition of active MMP through cation chelation or inhibition of oxidative activation of latent MMP [13, 14]. By inhibiting MMPs and reactive oxygen species, SDD protects the a1-proteinase inhibitor and, thus, indirectly reduces tissue proteinase activity [13, 14]. In addition, SDD stimulates the production of fibroblast collagen, reduces osteoclast activity and bone resorption, and directly influences the proliferation and differentiation of osteoprogenitor cells [15]. On the other hand, SDD does not alter oral microbiota and does not lead to antibiotic resistance [16].

Therefore, this study was conducted to evaluate the effect of the SDD as an adjunct to SRP on the GCF levels of MMP-9 and MMP-13 in type 2 diabetic subjects with periodontitis stage 2, grade B.

## 2. Materials and Methods

### 2.1. Subject Selection

This study included 60 periodontal sites within thirty type 2 diabetic patients (17 males and 13 females) with age ranging from 36 to 48 years. Patients were selected from individuals seeking care for periodontal conditions at the Department of Periodontology, Faculty of Dentistry, King Abdulaziz University, Saudi Arabia.

Patients were diagnosed with periodontitis stage 2, grade B according to Papapanou et.,al [2]. Inclusion criteria included (1) clinical loss of attachment (CAL) of 3-4 mm with radiographic bone loss limited to coronal third of the root. (2) No tooth loss due to periodontitis. (3) Maximum PD ≤ 5mm with mostly horizontal bone loss. (4) Grade B: indirect evidence of progression of 0.25 to 1.0 mm. The destruction is commensurate with biofilm deposits. (5) Shows grade modifiers; diagnosis with T2DM, with levels < 7.0% of (HbA1c).

Whereas the exclusion criteria were as follows: (1) patients were free from any other systemic conditions that affect the periodontium or interfere with the periodontal treatment except type 2 diabetes according to the modified Cornell Medical Index [17], (2) smokers and patients taking any medication that may affect soft and hard tissue healing, (3) pregnant as well as lactating mothers, and (4) previous periodontal surgery or antimicrobial therapy in the six months prior to our study.

All patients were given information regarding the proposed treatment and were asked to sign a consent form approved by the local ethics committee of King Abdulaziz University to voluntarily participate in this study (Protocol number: 101-10-18).

Each patient was asked to pick an envelope from several opaque sealed envelopes after fulfillment of the inclusion criteria and signing the informed consent to be enrolled in the study. The envelope contained the group to which the selected patient was allocated.

Following clinical examination and periapical radiographs, all participants were given detailed instructions in self-performed plaque control measures. The proposed nature of the study was explained. All patients received full mouth SRP using an ultrasonic scaler and hand instruments under local anesthesia to minimize pain.

A total of thirty patients were divided into the following two groups with reference to periodontitis, type 2 diabetes mellitus, and administration of doxycycline drug: Group I: included 15 patients with periodontitis stage 2, grade B and type 2 diabetes mellitus who received SRP only. Group II: included 15 patients with stage 2, grade B periodontitis and type 2 diabetes mellitus who received SRP and doxycycline 20 mg (doxycycline capsules, Adwaia Pharmaceutical Company) twice per day for three months.

Two periodontal sites were selected from each patient who fulfilled the inclusion criteria. Each patient's periodontal status was evaluated by measuring the Plaque Index (PI) [18], Gingival Index (GI) [19], and probing depth (PD) [20]. PD measurements were performed by a blinded examiner using a graduated periodontal probe (William's probe) and recorded to the nearest millimeter. The deepest PD was selected for each tooth per periodontal sites. The following measurements were recorded at baseline, then at one and three months.

Gingival clavicular fluid was collected at 0 (baseline), 1 month, and 3 months to assess matrix metalloproteinase-9 and metalloproteinase-13 levels. The collected samples were analyzed using enzyme-linked immunosorbent assay (ELISA).

The GCF was collected by placing the strips of periopaper in the selected periodontal site touching the base of the gingival sulcus. Each strip of paper was left for 30 seconds and then stored at -70°C in a sterile Eppendorf tube until analyzed. Samples were analyzed to determine MMP-13 and MMP-9 levels using a commercially available enzyme-linked immunosorbent assay kit (ELISA) (MMP-9 kit and MMP-13 ELISA kit purchased from Bioneovan Inova Co. Beijing, China). Before use, all reagents were allowed to warm to room temperature (18°C to 25°C). All standards and samples will be executed at least in duplicate. A total of 100 ml of anti-MMP9 or MMP-13 antibodies were added to each well and incubated for 1.5 hours. The solution was discarded and the wells were washed five times with wash solution (200 ml each). Then, 100 ml of each standard, positive control, and sample in the appropriate wells was added. The wells were covered and incubated for 2.5 hours at room temperature. The solution was discarded, and the wells were washed five times with wash solution (200 ml each). A total of 50 *μ*l of a prepared chromogen solution was added to each well and incubated for 15 minutes at room temperature. The solution was discarded, and the wells were washed five times with wash solution (200 *μ*l each). A total of 100 ml of tetramethylbenzidine substrate reagent was added in one step to each well and incubated for 30 minutes at room temperature in the dark. A total of 50 *μ*l of stop solution was added to each well. At 450 nm using a microtiter plate reader, the optical density value of the blank control well is set to zero. The test should be carried out within 15 minutes after adding the stop solution as the primary wavelength. The concentration calculation in each sample was performed by dividing the amount of MMPs by the volume of the sample (nanograms per milliliter).

### 2.2. Statistical Analysis

The collected data were tabulated and statistically analyzed using statistical program SPSS version 16.0 (Statistical Package for Social Sciences, SPSS, Inc., Chicago, IL, USA). ANOVA test was used to test the effect of the group on different measurements within each interval. Independent *t*-test was run to test the effect of intervals on different measurements within each group.

According to analyses of the mean probing depth, sample size calculation was undertaken via GPower version 3.1 statistical software: an alpha-type error of 0.05, a power test of 0.95, and effect size used in calculation: *d* = 1.3080913, entry 1 (3.00 ± 0.47) and entry 2 (3.48 ± 0.22), paired *t* was used.

Sample size calculations were achieved based on a previous study by Mastromatteo-Alberga et al. [21]. A total sample size of 14 patients (7 in each group) was sufficient to detect the difference based on this calculation. However, the present study was carried out on fifteen patients with thirty periodontal defects in each group.

## 3. Results

This study was conducted on thirty patients. They included 17 males and 13 females with an age range from 36 to 48 years. All patients have the criteria of stage 2, grade B periodontitis with T2DM. All 30 patients completed treatment and had no adverse reactions to therapy.

### 3.1. Clinical Parameters

#### 3.1.1. Gingival Index

**Figure 1:**
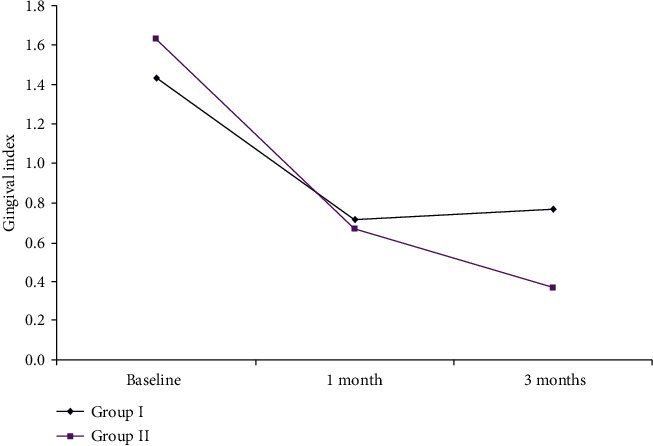
The mean GI reduction in Group I and Group II.

The mean GI reduction in the two groups is shown in Figure 1 and Table 1.

In Group I, there was a reduction in GI by −0.73 ± 0.45 and −0.67 ± 0.55 at one and 3 months. On the other hand, Group II showed a decrease in GI by −0.97 ± 0.41 and −1.27 ± 0.74 at one and 3 months, respectively, compared to baseline measurements. Statistical analysis regarding mean change in GI showed statistically significant differences in GI reduction at one and 3 months, respectively, between both groups.

#### 3.1.2. Plaque Index

**Figure 2:**
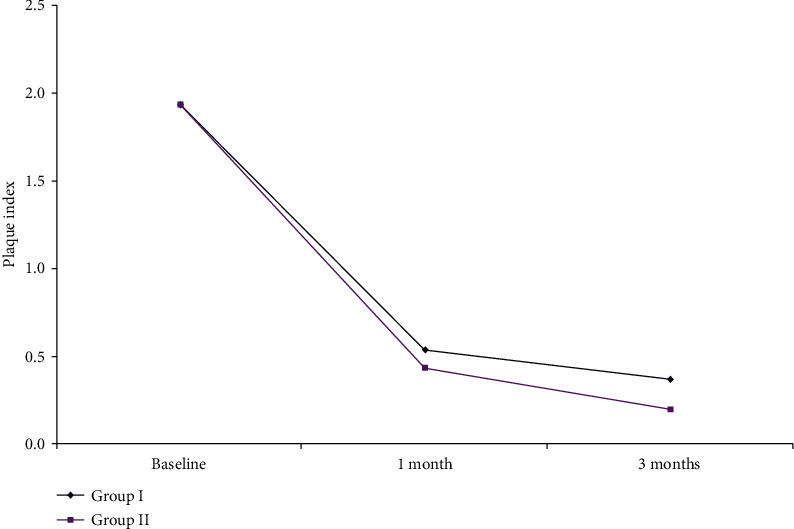
The mean PI changes in Group I and Group II.

As shown in Figure 2 and Table 1, the mean PI results within each Group I and II revealed significant reduction changes at baseline, one month, and 3 months. However, statistically nonsignificant differences were observed on comparing one- and 3-month results within both groups.

#### 3.1.3. Probing Depth

**Figure 3:**
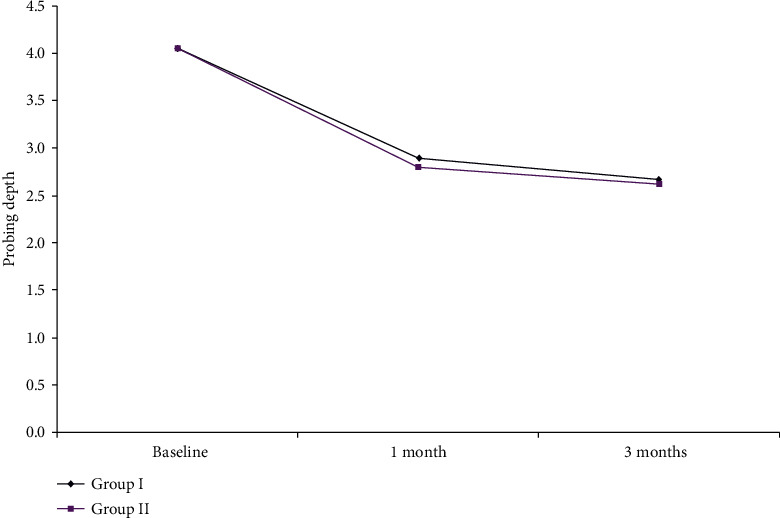
The mean probing depth changes in Group I and Group II.

The mean PD results within Group I are shown in Figure 3 and Table 1. There was a significant reduction throughout the study period in each group. There was a nonsignificant difference between the baseline, one-, and 3-month readings between both groups. However, maximum reduction of PD in Group I reached −1.37 ± 0.61 mm while Group II showed more reduction in PD measurements which reached −1.40 ± 0.50 mm at the end of the study period (*P* ≤ 0.001).

### 3.2. Biochemical Results

#### 3.2.1. Matrix Metalloproteinase-9

MMP-9 concentrations within both Group I and Group II showed a statistically significant decrease in measurement between the baseline and one and 3 months (Table 2). The mean percent decrease in MMP-9 for Group I was −347.24 ± 183, while it was −770.11 ± 308.88 for Group II from baseline to one month. The difference between the both groups in the change in MMP-9 from baseline to 3 months posttreatment was statistically significant (*P* ≤ 0.001). (Table 1).

#### 3.2.2. Matrix Metalloproteinase-13

Table 3 illustrated mean concentrations of MMP-13 within Groups I and II. In Group II, maximum reduction in MMP-13 was noticed (−1076.83 ± 390.94) while the total reduction in MMP-13 in Group I was (−968.33 ± 609.19) after 3 months. A significant difference was illustrated in the decrease in MMP-13 between both groups at one month and 3 months, respectively.

## 4. Discussion

Periodontal disease can be treated successfully by nonsurgical or surgical mechanical approach to reduce tissue inflammation. Some investigators suggest that the levels of proinflammatory markers increase when localized and/or systemic inflammatory processes occur [22]. Thus, the evaluation of MMP levels before and after periodontal treatment may provide a mechanism to monitor host response in periodontal disease and evaluate treatment outcome.

Chee et al. [23] suggest that patients with type 2 diabetes are more likely to have a greater severity of periodontal disease. They demonstrated that diabetic subjects who received periodontal treatment maintain control of their metabolic levels. On the other hand, Rajhans et al. [24] observed the abnormal lymphocyte function as a result of hyperglycemia, which also leads to the formation of advanced glycosylated end products and finally to early cell apoptosis in response to high MMP levels.

In this study, subjects who took SDD exhibited a significantly greater reduction in PD and GI and a reduction in GCF MMP-9 and MMP-13 levels than those who received SRP alone. This improvement could be attributed not only to the SRP and to the appropriate oral hygiene measures maintained by the patient but also to the modulating effect of the SDD on the periodontal tissues, where SDD has anti-inflammatory and anticollagenase effects that improve the healing of periodontal tissues [25].

A previous study demonstrated the efficacy of SDD in dampening microbial-induced gingival inflammation in patients with DM and advanced periodontal loss [26]. Similarly, a greater reduction in GI was observed notably in Group II that received the SDD protocol for three months. These findings support the conception that SDD has anti-inflammatory, antiprololytic effects and can suppress angiogenesis [27].

Several studies [28–30] demonstrated a relationship between MMP and connective tissue loss. Growth factors, cytokines, different MMPs, and components of the extracellular matrix can regulate MMPs during inflammatory stimulation. For example, MMPs are capable of degrading ECM. This degradation is induced by proinflammatory cytokines such as IL-1*β* and TNF*α*, suggesting that these enzymes play an important role in the pathogenesis of periodontitis [31].

MMP production during the acute phase of periodontitis is elevated by IL-1 or IL-6 produced by neutrophils [32]. In addition, several MMPs are linked together where MMP-13 could be involved in the degradation of soft and hard support tissues and the activation of pro-MMP-9 during the progression of chronic periodontitis. Interestingly, MMP-13 and MMP-9 can potentially form an activation cascade that exceeds the TIMP-1 protective shield, which can be useful for diagnostic purposes and an objective for drug development [33].

Statistical analysis between both groups with respect to the mean change in MMP-13 concentration showed that nonsignificant differences were observed between the first and the second interval. Also, Group I revealed a greater reduction in MMP-13 at the end of the study period. On the other hand, a statistically significant difference was observed in each interval between Group I and Group II in the average change in MMP-9. The greatest reduction in Group II that received SDD could be attributed to the action of the drug that inhibits the destruction of connective tissue through the direct inhibition of MMPs in gingival tissues and inhibits the activation of MMP precursors [34, 35].

The clinical and biochemical results of the present study were in agreement with Tuter et al. [36] that showed statistically significant differences in GI, PD, and GCF levels of MMP-8 between groups favoring the group receiving SDD adjunctive to SRP

In addition, in a randomized, double-blind, controlled study conducted by Gilowski et al. (2012), the effectiveness of short-term SDD was studied as an adjunct to SRP in patients with DM2 and periodontitis by evaluating MMP-8 levels in the GCF. The results revealed a statistically significant reduction in GCF MMP-8 levels only in the test goup that received SDD for 3 months [37].

## 5. Conclusion

In conclusion, the use of subantimicrobial dose doxycycline with nonsurgical periodontal therapy resulted in favorable clinical and biochemical results in periodontitis stage 2, grade B in subjects with type 2 diabetes mellitus. Periodontal defects in which therapy combined with SDD (Group II) demonstrated superior results than the defects in which SRP therapy was used alone (Group I). This represents an important clinical advantage for patients with type 2 diabetes mellitus. Diabetic patients should also maintain their glycemic control and continue their oral hygiene measures. In addition, the expression of MMP-9 and MMP-13 could be relevant predictive indicators for the progression of periodontitis.

Further controlled and prospective studies are needed to study the effects of the subantimicrobial dose doxycycline as adjunctive aids to the nonsurgical approach of periodontal therapy in diabetic patients utilizing different biological markers. In addition, more investigations on the triggering mechanisms of destruction carried by MMP-9 and MMP-13 are needed.

## Figures and Tables

**Table 1:** The reduction in different clinical and biochemical parameters in both groups.

Mean differences		Group I	Group II	Test value	*P* value	Sig.
		Mean ± SD	Mean ± SD			
Gingival index	1 month-baseline	−0.73 ± 0.45	−0.97 ± 0.41	-2.159	0.035	S
	3 months-baseline	−0.67 ± 0.55	−1.27 ± 0.74	-3.564	≤0.001	HS
Plaque index	1 month-baseline	−1.40 ± 0.56	−1.50 ± 0.51	-0.723	0.473	NS
	3 months-baseline	−1.57 ± 0.57	−1.73 ± 0.52	-1.136	0.261	NS
Probing depth	1 month-baseline	−1.13 ± 0.51	−1.23 ± 0.43	-0.821	0.415	NS
	3 months-baseline	−1.37 ± 0.61	−1.40 ± 0.50	-0.208	0.836	NS
MMP-9	1 month-baseline	−347.24 ± 183	−770.11 ± 308.88	-6.451	≤0.001	HS
	3 months-baseline	−572.58 ± 303.28	−999.62 ± 396.83	-4.683	≤0.001	HS
MMP-13	1 month-baseline	−181.67 ± 69.73	−257 ± 177.38	-2.165	0.035	S
	3 months-baseline	−968.33 ± 609.19	−1076.83 ± 390.94	-0.821	0.415	NS

**Table 2:** The change in mean MMP-9 conc. at different intervals.

	MMP-9			Test value^a^	*P* value	Sig.
	Baseline	1 month	3 months			
	Mean ± SD	Mean ± SD	Mean ± SD			
Group I	1088.41 ± 523.45	741.17 ± 340.45	515.83 ± 220.17	17.081	≤0.001	HS
Group II	1330.45 ± 539.33	560.34 ± 230.45	330.83 ± 142.50	67.734	≤0.001	HS
Test value^b^	1.764	-2.409	-3.864			
*P* value	0.083	0.019	≤0.001			

*P* value > 0.05: nonsignificant; *P* value < 0.05: significant; *P* value < 0.01: highly significant. ^a^Repeated measure ANOVA test; ^b^independent *t*-test.

**Table 3:** The change in mean MMP-13 conc. at different intervals.

	MMP-13			Test value^a^	*P* value	Sig.
	Baseline	1 month	3 months			
	Mean ± SD	Mean ± SD	Mean ± SD			
Group I	1798.00 ± 1047.49	1616.33 ± 977.76	829.67 ± 438.30	10.619	≤0.001	HS
Group II	1393.00 ± 714.03	1136.00 ± 891.41	316.17 ± 323.09	20.205	≤0.001	HS
Test value^b^	-1.750	-1.988	-5.165			
*P* value	0.085	0.052	≤0.001			

*P* value > 0.05: nonsignificant; *P* value < 0.05: significant; *P* value < 0.01: highly significant. ^a^Repeated measure ANOVA test; ^b^independent *t*-test.

## Data Availability

The data used to support the findings of this study are available from the corresponding author upon request.

## References

[1] Chaffee B. W., Weston S. J. (2010). Association between chronic periodontal disease and obesity: a systematic review and meta-analysis. *Journal of Periodontology*.

[2] Papapanou P. N., Sanz M., Buduneli N. (2018). Periodontitis: Consensus report of workgroup 2 of the 2017 World Workshop on the Classification of Periodontal and Peri-Implant Diseases and Conditions. *Journal of Periodontology*.

[3] Preshaw P. M., Taylor J. J. (2011). How has research into cytokine interactions and their role in driving immune responses impacted our understanding of periodontitis?. *Journal of Clinical Periodontology*.

[4] Dandona P., Aljada A., Bandyopadhyay A. (2004). Inflammation: the link between insulin resistance, obesity and diabetes. *Trends in Immunology*.

[5] Brownlee M. (2005). The pathobiology of diabetic complications: a unifying mechanism. *Diabetes*.

[6] Molina C. A., Ojeda L. F., Jiménez M. S. (2016). Diabetes and Periodontal Diseases: An Established Two-Way Relationship. *Journal of Diabetes Mellitus*.

[7] Kim K.-A., Chung S.-B., Hawng E.-Y. (2013). Correlation of expression and activity of matrix metalloproteinase-9 and -2 in human gingival cells of periodontitis patients. *Journal of Periodontal & Implant Science*.

[8] Rathnayake N., Gustafsson A., Norhammar A. (2015). Salivary Matrix Metalloproteinase-8 and -9 and Myeloperoxidase in Relation to Coronary Heart and Periodontal Diseases: A Subgroup Report from the PAROKRANK Study (Periodontitis and Its Relation to Coronary Artery Disease). *PLOS ONE*.

[9] Hernández M., Dutzan N., García-Sesnich J. (2011). Host-Pathogen Interactions in Progressive Chronic Periodontitis. *Journal of Dental Research*.

[10] Hernandez M., Valenzuela M. A., Lopez-Otin C. (2006). Matrix metalloproteinase-13 is highly expressed in destructive periodontal disease activity. *Journal of Periodontology*.

[11] Drisko C. H. (2001). Nonsurgical periodontal therapy. *Periodontology 2000*.

[12] Eley B. M., Manson J. D. (2004). *Periodontics*.

[13] Kaur M., Kumar K. (2017). Subantimicrobial dose doxycycline in treatment of periodontitis. *International Journal of Applied Dental Sciences*.

[14] Golub L. M., Lee H.-M., Ryan M. E., Giannobile W. V., Payne J., Sorsa T. (2016). Tetracyclines Inhibit Connective Tissue Breakdown by Multiple Non-Antimicrobial Mechanisms. *Advances in Dental Research*.

[15] Gomes P. S., Fernandes M. H. (2007). Effect of therapeutic levels of doxycycline and minocycline in the proliferation and differentiation of human bone marrow osteoblastic cells. *Archives of Oral Biology*.

[16] Thomas J., Walker C., Bradshaw M. (2000). Long-term use of subantimicrobial dose doxycycline does not lead to changes in antimicrobial susceptibility. *Journal of Periodontology*.

[17] Kerr H. D., Ash D. A., Millard M. (1965). *Oral Diagnosis*.

[18] Silness J., Löe H. (1964). Periodontal Disease in Pregnancy II. Correlation Between Oral Hygiene and Periodontal Condition. *Acta Odontologica Scandinavica*.

[19] Löe H., Silness J. (2009). Periodontal Disease in Pregnancy I. Prevalence and Severity. *Acta Odontologica Scandinavica*.

[20] Polson A. M., Caton J. G., Yeaple R. N., Zander H. A. (1980). Histological determination of probe tip penetration into gingival sulcus of humans using an electronic pressure-sensitive probe. *Journal of Clinical Periodontology*.

[21] Mastromatteo-Alberga P., Instituto de Investigaciones Odontológicas Raul Vincentelli, Facultad de Odontología (2018). Cytokines and MMPs levels in gingival crevicular fluid from patients with chronic periodontitis before and after non-surgical periodontal therapy. *Journal of Oral Research*.

[22] Sorsa T., Mantyla P., Tervahartiala T., Pussinen P. J., Gamonal J., Hernandez M. (2011). MMP activation in diagnostics of periodontitis and systemic inflammation. *Journal of Clinical Periodontology*.

[23] Chee B., Park B., Bartold M. P. (2013). Periodontitis and type II diabetes: a two-way relationship. *International Journal of Evidence-Based Healthcare*.

[24] Rajhans N. S., Chaudhari V. G., Kohad R. M., Mhaske N. H. (2011). A clinical study of the relationship between diabetes mellitus and periodontal disease. *Journal of Indian Society of Periodontology*.

[25] Walker C., Thomas J., Nango S., Lennon J., Wetzel J., Powala C. (2000). Long-term treatment with subantimicrobial dose doxycycline exerts no antibacterial effect on the subgingival microflora associated with adult periodontitis. *Journal of Periodontology*.

[26] Deo V., Gupta S., Bhongade M. L., Jaiswal R. (2010). Evaluation of subantimicrobial dose doxycycline as an adjunct to scaling and root planing in chronic periodontitis patients with diabetes: a randomized, placebo-controlled clinical trial. *The Journal of Contemporary Dental Practice*.

[27] Garrido-Mesa N., Zarzuelo A., Galvez J. (2013). What is behind the non-antibiotic properties of minocycline?. *Pharmacological Research*.

[28] Corotti M. V., Zambuzzi W. F., Paiva K. B. S. (2009). Immunolocalization of matrix metalloproteinases-2 and -9 during apical periodontitis development. *Archives of Oral Biology*.

[29] Emingil G., Kuula H., Sorsa T., Atilla G. (2006). Gingival crevicular fluid matrix metalloproteinase-25 and -26 levels in periodontal disease. *Journal of Periodontology*.

[30] Nishikawa M., Yamaguchi Y., Yoshitake K., Saeki Y. (2002). Effects of TNFalpha and prostaglandin E2 on the expression of MMPs in human periodontal ligament fibroblasts. *Journal of Periodontal Research*.

[31] Shapiro S. D., Senior R. M. (1999). Matrix Metalloproteinases. *American Journal of Respiratory Cell and Molecular Biology*.

[32] Márton I. J., Kiss C. (2000). Protective and destructive immune reactions in apical periodontitis. *Oral Microbiology and Immunology*.

[33] Ríos M. H., Sorsa T., Obregón F. (2009). Proteolytic roles of matrix metalloproteinase (MMP)-13 during progression of chronic periodontitis: initial evidence for MMP-13/MMP-9 activation cascade. *Journal of Clinical Periodontology*.

[34] Golub L. M., Evans R. T., McNamara T. F., Lee H. M., Ramamurthy N. S. (1994). A Non-Antimicrobial Tetracycline Inhibits Gingival Matrix Metalloproteinases and Bone Loss in Porphyromonas gingivalis-induced Periodontitis in Rats. *Annals of the New York Academy of Sciences*.

[35] Rifkin B. R., Vernillo A. T., Golub L. M. (1993). Blocking Periodontal Disease Progression by Inhibiting Tissue-Destructive Enzymes: A Potential Therapeutic Role for Tetracyclines and Their Chemically-Modified Analogs. *Journal of Periodontology*.

[36] Tüter G., Serdar M., Kurtiş B. (2010). Effects of Scaling and Root Planing and Subantimicrobial Dose Doxycycline on Gingival Crevicular Fluid Levels of Matrix Metalloproteinase-8, -13 and Serum Levels of HsCRP in Patients With Chronic Periodontitis. *Journal of Periodontology*.

[37] Gilowski L., Kondzielnik P., Wiench R., Plocica I., Strojek K., Krzeminski T. F. (2012). Efficacy of short-term adjunctive subantimicrobial dose doxycycline in diabetic patients--randomized study. *Oral Diseases*.

